# The New Global Health

**DOI:** 10.3201/eid1908.130121

**Published:** 2013-08

**Authors:** Kevin M. De Cock, Patricia M. Simone, Veronica Davison, Laurence Slutsker

**Affiliations:** Centers for Disease Control and Prevention, Atlanta, Georgia, USA

**Keywords:** public health, global health, HIV/AIDS and other retroviruses, viruses, bacteria, parasites, tuberculosis, malaria, neglected topical diseases, dengue, yellow fever, cholera, typhoid, hepatitis, polio, meningitis, influenza, global health development, infectious diseases, health security

## Abstract

Global health reflects the realities of globalization, including worldwide dissemination of infectious and noninfectious public health risks. Global health architecture is complex and better coordination is needed between multiple organizations. Three overlapping themes determine global health action and prioritization: development, security, and public health. These themes play out against a background of demographic change, socioeconomic development, and urbanization. Infectious diseases remain critical factors, but are no longer the major cause of global illness and death. Traditional indicators of public health, such as maternal and infant mortality rates no longer describe the health status of whole societies; this change highlights the need for investment in vital registration and disease-specific reporting. Noncommunicable diseases, injuries, and mental health will require greater attention from the world in the future. The new global health requires broader engagement by health organizations and all countries for the objectives of health equity, access, and coverage as priorities beyond the Millennium Development Goals are set.

“People are beginning to understand there is nothing in the world so remote that it can’t impact you as a person.”—William H. Foege, Director, US Centers for Disease Control, 1977–1983

Health has become an area for diplomatic engagement and a priority subject on the world stage. Funding for global health has reached ≈$30 billion/year, and the United States provides at least one third of this total (*1*). However, too often there is lack of coordination across the inordinately complex architecture of global health. Agencies other than the World Health Organization (WHO), such as the World Bank and the Bill and Melinda Gates Foundation, have become prominent funders that influence policy; new multilateral organizations, such as the United Nations Joint Programme on HIV/AIDS, the Global Alliance for Vaccines and Immunisation, UNITAID, and the Global Fund to Fight AIDS, Tuberculosis and Malaria have sprung up; and civil society groups such as Médecins Sans Frontières implement programs and exert substantial political pressure.

These developments have challenged WHO, which although retaining unique credibility and convening authority, is hampered by funding shortages and donor-imposed earmarks, an inflexible bureaucratic and governance structure, and difficulty prioritizing in the face of unrealistic demands. Many decisions are now made outside the World Health Assembly, the world’s senior and most representative forum for global health discussion. Newer global health actors are often seen as swifter and more focused on performance and accountability.

With global emphasis on austerity, there is now more than ever a need for bilateral and multilateral assistance to be coordinated for maximal effect, to avoid duplication and gaps, and to focus on measureable results. The diversity of multilateral agencies working in health distracts from the limited essentials expected from the global sector: estimating fiscal requirements and tracking financing; normative guidance; detecting and coordinating responses to complex emergencies and international health threats; monitoring and communicating health trends; and advocacy. A first requirement, including for bilateral partners, is agreement on what constitutes global health and which agencies are best placed to play particular roles.

This report discusses the evolving nature of global health and its priorities. Progress requires revision of the dichotomous view of a static world of industrialized or developing countries, rich or poor. Today’s health disparities are as extreme within countries as between them. A more useful perspective is that global health requires synergistic engagement by all countries in an interdependent world, replacing the model of donors and recipients that characterized earlier international health assistance.

## Global Health

The term global health has replaced tropical medicine and international health, disciplines linked to the history of colonialism, the post-independence era of the former European colonies, and the experience of development assistance (*2*,*3*). Global health is multidisciplinary, encompasses many elements besides development, and requires coordination of multiple parties, rather than direction by one organization or discipline. The increased technical and political complexity of global health, with many actors, including philanthropic and faith-based organizations, is reflected in its breadth, which covers diverse diseases but deals also with health systems issues and financing.

Global health reflects the realities of globalization, especially the increased movement of persons and goods, and the global dissemination of infectious and noninfectious public health risks. Global health is concerned with protecting the entire global community, not just its poorest segments, against threats to health and with delivering essential and cost-effective public health and clinical services to the world’s population. A fundamental tenet is that no country can ensure the health of its population in isolation from the rest of the world, as articulated in the Global Health Strategy of the United States Department of Health and Human Services (*4*). This vision reflects today’s health realities but was arrived at through milestones such as the 1993 World Development Report (Investing in Health) (*5*), the 2000 report of the Commission on Macroeconomics and Health (*6*), and the tremendous investment in HIV/AIDS begun earlier this century (*7*).

## Development, Security, and Public Health

Three overlapping themes determine global health action: development, security, and public health. These themes provide the humanitarian and political bases for engagement by high-income countries in health matters internationally: for development, to promote health for stability, prosperity, and better international relationships; for security, to protect their populations against internal and external health threats; and for public health, to save lives worldwide and at home. Despite different requirements, organizations and agencies involved must adapt to global trends in socioeconomic development, fertility, population, and urbanization.

### Development

Of 214 countries categorized by the World Bank, only 36 (17%) were classified as low-income countries (gross national income per capita in 2011 <$1,025 per year), 26 of which were in Africa (*8*). Economic growth is moving some low-income countries toward middle-income status, and some of the greatest imbalances in wealth may now be within rather than between individual countries. With socioeconomic development, basic health indicators improve but so do countries’ abilities to shoulder more of their own health expenditures. Several middle-income countries such as the BRICS (Brazil, Russia, India, China, South Africa), and countries with oil-rich economies are capable of delivering assistance to poorer nations.

A clear correlation exists between countries’ gross domestic product and their health indicators, such as mortality rates in children <5 years of age (highest in low-income countries) or life expectancy (highest in high-income countries). Development raises living standards, accompanied by improvement in basic services and drivers of health, such as nutrition and food security; access to potable water and sanitation; maternal and child health interventions, including family planning; and basic education, especially for women. The fundamental responsibility for development agencies, and their greatest contribution to health, is poverty reduction.

Although family planning and maternal and child health remain high on the development agenda, demographic trends are changing rapidly. Since 1980, the world’s population has increased by nearly 60%; from ≈7 billion today, global population is projected to reach 9.3 billion by 2050 and 10.1 billion by 2100 (*9*). Decreasing fertility trends in sub-Saharan Africa are now following a similar trajectory as occurred elsewhere, but separated by several decades. By the end of the twenty-first century, the population of Africa will likely have increased by ≈2.6 billion, compared with ≈432 million in Asia. The Democratic Republic of Congo, Ethiopia, and Nigeria will be new demographic giants; it is predicted that in 2100, Nigeria will have a population of 730 million persons (*9*). By 2025 more than half of the world’s citizens will live in urban settings, with dozens of megacities characterized by populations >10 million persons, including many in Africa (*9*), all challenged by the need for basic infrastructure and services.

A welcome trend has been renewed attention to reducing avoidable deaths among children. The worldwide reduction in childhood mortality rates means that since the 1980s, deaths among adults have exceeded deaths among children. Recently published estimates of mortality rates among children <5 years of age indicate that there are ≈7.2–7.6 million childhood deaths/year compared with ≈12 million deaths only 2 decades ago (*10*–*12*). Since 1990, maternal deaths have decreased from ≈526,000 to ≈274,000 (*11*).

Six countries, each with >200,000 deaths annually among children <5 years of age, account for ≈50% of global deaths in children; >50% of deaths in children occur in sub-Saharan Africa. Because of their large populations, India and China contribute substantially to these deaths, as do large countries with poor health indicators, such as Nigeria, the Democratic Republic of Congo, Pakistan, and Ethiopia. Seven countries with >10,000 maternal deaths/year account for >50% of the world’s maternal mortality rate. The highest maternal mortality rates are in sub-Saharan Africa, especially western Africa, a finding that is consistent with distribution of adverse rates of child survival. Pakistan and Afghanistan stand out for unfavorable indicators in their region. Further reduction in maternal and child mortality rates globally will require special focus on countries with the greatest absolute numbers of maternal and child deaths.

### Health Security

Drawing on earlier United Nations perspectives that characterized poor health as one of several threats to human security and well-being, health security captures the need for collective action and preparedness to reduce vulnerabilities to public health threats that transcend borders (*13*). Earlier optimism predicting the end of infectious diseases was replaced by recognition of the threat to global health from emerging infectious diseases and widespread antimicrobial drug resistance (*14*). The pandemic of HIV/AIDS, repeated outbreaks of Ebola and Marburg virus infections, rapid international dissemination of severe acute respiratory syndrome and pandemic influenza, international spread of several foodborne pathogens, and the intentional transmission of anthrax all convincingly illustrated global vulnerability. Other aspects of globalization negatively affecting health security include the trafficking of drugs and persons and population movement consequent to conflict and instability.

The global framework for health security is embodied in the International Health Regulations that were revised in 2005 and adopted by the World Health Assembly, but whose implementation is lagging behind the 2012 target date (*15*). The diversity of health threats results in involvement of other sectors, such as defense and diplomacy, and linkage with other international agreements, such as those relating to control of chemical, biological, and nuclear weapons.

Surveillance and laboratory capacity through strong national public health institutes are essential components of functioning health systems that provide the basis for health security. Ensuring ability to detect, investigate, diagnose, and rapidly contain public health events of concern wherever they occur requires commitment to global health capacity development in all countries and widespread and supportive public health networks (*16*).

### Public Health

The scale-up of programs for HIV/AIDS, malaria, and tuberculosis over the past decade through initiatives such as the Global Fund, the United States President’s Emergency Plan for AIDS Relief, and the President’s Malaria Initiative led to substantial disease-specific progress. The Global Alliance for Vaccines and Immunisation has positively affected vaccine access. However, these experiences also highlighted the relative neglect of other priority areas and led to criticism that vertical, targeted programs failed to strengthen health systems overall (*17*). As a result, there has been renewed focus on the other health-related Millennium Development Goals (MDGs), especially relating to children’s and maternal health (MDGs 4 and 5, respectively). These perceptions contributed to the establishment of the United States government’s Global Health Initiative in 2009 (*18*) that addresses all health MDGs and some neglected tropical diseases in a more integrated manner.

The longstanding tension between vertical and horizontal approaches is now better understood, and there is greater emphasis on integration of efforts (*19*). Initiatives to strengthen general health systems have lacked specificity and agreed upon indicators, and they have had more difficulty showing measurable effects than disease-specific interventions that emphasize integration and linkage to other services. Public health agencies have a major role in strengthening specific areas of health systems, such as health information systems and surveillance, laboratory capacity, workforce skills, operational research and evaluation, and capacity for preparedness and program implementation (*20*).

National public health institutes and strong ministries have the core responsibility for defining policies, goals and targets, and assuring technical guidance, supervision, program implementation, evaluation, and accountability (*21*). Although epidemiology remains at the core of such work, the increased complexity of combinations of interventions in public health has highlighted the utility of mathematical modeling for assisting in decision making and policy setting.

Modern public health agencies have to be global in outlook to fulfill their domestic mandates. Because of the credibility emanating from their technical expertise, these agencies play an essential role in health diplomacy and development of public health capacity. Although development agencies concentrate on the needs of the poor, public health agencies potentially interact with all countries to address common challenges. Health systems strengthening, communicable and noncommunicable disease threats, safety and quality of medicines and commodities, and health access and equity are universally challenging to ministries of health, public health institutes and multilateral organizations, which all need to function in a close global network.

## Unfinished Business: Infectious Disease Priorities

Recent estimates of the global incidence of disease suggest that communicable diseases account for ≈19% of global deaths (*22*). In Africa, 76% of deaths are still attributable to communicable, maternal, neonatal, or nutritional causes, compared with 25% in the entire world; conditions relevant to MDGs 4, 5, and 6 are responsible for 42% of years of life lost. Focus on infectious diseases remains necessary to prevent their global spread or recrudescence, save lives, enhance economic development, and increase health equity.

Major and persistent infectious disease threats, their global incidence, and some of the global health commitments made to address them are shown in the Technical Appendix. The 1993 World Bank report Investing in Health first highlighted the overwhelming role of HIV/AIDS, tuberculosis, and malaria in Africa (*5*), but only in the past decade have substantially increased investment and effort enabled measurable progress in these major infectious disease challenges. The world needs to maintain momentum to achieve ambitious health targets and implement recent scientific advances while simultaneously coping with economic austerity.

There is increasing pressure to use resources for biomedical interventions with the strongest evidence of efficacy. Efforts toward achieving an AIDS-free generation are centered around HIV treatment scale-up, prevention of mother-to-child transmission (including through immediate and life-long antiretroviral therapy for all HIV-infected pregnant women), medical male circumcision, HIV testing and counseling, and focus on key populations in which HIV infection is concentrated (*23*). The primary current research question in HIV/AIDS is how best to use antiretroviral therapy for individual health and for population-based prevention, and more specifically, whether immediate therapy upon early diagnosis would confer the greatest benefit (*24*,*25*). The commitment to virtual elimination of HIV disease in children (*26*) could usefully link new initiatives to traditional maternal and child health programs delivered through development funding.

Tuberculosis is decreasing in incidence in all regions of the world, although more slowly than expected in some regions (*27*). In the United States, 63% of all tuberculosis cases now occur in foreign-born persons, indicating likely acquisition of the infection outside the United States (*28*). The spread of drug-resistant tuberculosis and extensively drug-resistant tuberculosis (resistant to rifampin, isoniazid, quinolones and injectable antituberculous drugs) highlights global vulnerability and interrelatedness of health systems and challenges health equity. Key scientific advances concern better understanding of the role and use of antiretroviral therapy for persons with tuberculosis co-infected with HIV, new diagnostics with the potential to make case finding more effective, and less strikingly, new drugs.

The tools for combating malaria (insecticide-impregnated bed nets, indoor residual spraying of insecticide, artemisinin-based combination therapies, and intermittent preventive therapy for pregnant women) need further scale-up, but such tools are susceptible to development of resistance on the part of the vector or parasite, and evidence is accumulating that nets may be less durable than assumed (*26*,*29*). Despite the challenges, malaria elimination has risen up the global agenda in recent time.

Poverty-related diseases such as the 17 conditions categorized as neglected tropical diseases have also received increased investment, especially those for which mass drug administration offers a control strategy (*30**,**31*). A concern must be that some major causes of illness and death, such as visceral leishmaniasis and African human trypanosomiasis, remain overshadowed and unaddressed.

Two groups of diseases meriting global health attention are those that are epidemic prone or vaccine preventable, including influenza. The 2009 pandemic of influenza A(H1N1) demonstrated the global nature of the threat, as well as the need to consider strategies for provision of vaccine for all countries (*32*). Dengue and yellow fever are the major mosquito-borne viral infections, and both illustrate the concept of emerging infections promoted by diverse factors, such as urbanization, population growth, inadequate environmental hygiene, and vector resistance to insecticide. In recent years, large outbreaks involving a specific arbovirus, chikungunya virus, have affected the east coast of Africa and islands in the Indian Ocean with importation into Europe.

The second decade of this century has been designated as the decade of vaccines (*33*). The opportunity exists for a notable effect on the 2.5 million deaths of children annually from vaccine-preventable diseases, including through use of new vaccines for prevention of rotavirus and pneumococcal infection, and by strengthening routine services. Vaccination against type A meningococcal meningitis in the Sahel and against hepatitis B virus and human papillomavirus illustrate the unrivaled possibilities in terms of controlling previously deadly epidemics or virus-induced cancers. A major unfinished priority is polio eradication; this goal is particularly threatened by funding shortfalls and ongoing transmission in Pakistan, Afghanistan, and Nigeria, which have seeded infection in other countries in which polio had been eliminated (*34*).

Lack of access to water and sanitation highlights some of the greatest inequities in global health. Approximately 1 billion persons worldwide do not have clean drinking water, and ≈2.5 billion persons have to openly defecate, which is an affront to human dignity (*35*). Large epidemics of waterborne diseases continue to occur, as exemplified by ongoing cholera transmission in Haiti (*36*). It is difficult to explain why investment in separating human drinking water from human feces, the basis of the nineteenth century public health revolution in Europe and North America, has not been a higher political or development priority in resource-poor settings.

## Noncommunicable Diseases

The high-level meeting on noncommunicable diseases at the General Assembly of the United Nations in 2011, only the second such meeting devoted to health, emphasized how these diseases now dominate health worldwide (*37*). More than 60% of preventable deaths worldwide are now attributable to noncommunicable diseases (cardiovascular diseases, cancers, diabetes, and chronic respiratory diseases); in low-income and middle-income countries, 48% of such deaths occur in persons <70 years of age, compared with 26% in high-income countries (*38*). The incidence of these conditions is also increasing rapidly in Africa, a region in which urbanization and population growth are most extreme.

The risk factors for noncommunicable diseases are associated with urbanization and altered lifestyles, especially smoking, physical inactivity, air pollution, unhealthy diet, and excessive alcohol use. Hypertension, obesity, and increased cholesterol levels are measurable indicators predicting adverse outcomes, and specific chronic infections, such as those with hepatitis B virus and human papillomavirus, are linked to certain cancers. Injuries and mental health were omitted from the 2011 United Nations agenda despite the increasing incidence of these conditions; each year >5 million deaths worldwide result from injuries and violence (*39*), and ≈1.3 million are caused by road traffic injuries. Mental and behavioral disorders are considered the largest contributor to years lived with disability (*22*).

Global funding for noncommunicable diseases is minimal and coordination is limited, although opportunities exist for integrating approaches to communicable and noncommunicable diseases. Implementation of surveillance to assess incidence and needs along with selected policy interventions to address them will have the greatest immediate effect for the least cost. Examples of such policies include restricting tobacco sales and access, raising tobacco taxes, limiting unsafe use of alcohol, enacting motorcycle helmet and seat belt laws, and reducing salt and trans fats in commercial food products. To encourage countries to take action, WHO is defining population-level targets for noncommunicable diseases and associated risk factors for program implementation (*37*). Experience with HIV/AIDS treatment scale-up (*40*) could provide useful lessons for a standardized approach to management of hypertension and diabetes, thereby enhancing cost-effectiveness; facilitating supervision, monitoring, and evaluation; and ensuring accountability.

## Conclusions

Population growth, increased life expectancy of the world’s citizens, and decreased age-specific mortality rates in children and young adults, especially those for infectious diseases, have contributed to the altered global health landscape. The New Global Health concerns health in all countries and encompasses poverty alleviation, universal health security, and delivery of appropriate public health and clinical services, including for the increasing prevalence of noncommunicable diseases.

Equity, universal health coverage and access, and fairness in health financing are global aspirations likely to feature prominently in discussions about what comes after the 2015 MDG target date. The unfinished infectious disease agenda will remain a priority, but common approaches will have to address noncommunicable diseases, regulation of commerce in medical technologies and pharmaceuticals, health financing, and systems strengthening. An emerging topic will be surveillance for and mitigation of effects of environmental and climate change.

Surveillance will have to be strengthened globally to track exposure to risk factors for the major causes of disability and death, disease outcomes, and health systems responses. The past and on-going epidemiologic transitions mean that in many countries, the classic health indicators of international health (infant, under-5, and maternal mortality rates) no longer provide insight into population health. In addition, there is an urgent need for robust vital registration systems and accurate reporting of cause-specific mortality rates across all life stages.

We must not forget the current challenges facing the lowest-income countries, the needs of disenfranchised or displaced populations, societies threatened by conflict and humanitarian emergencies, and the urban and rural poor living conditions in the midst of plenty. Nonetheless, global health practice must adapt to globalization and the rapid evolution in health underway worldwide. For donor countries, this will require clear definition of expectations of development assistance and how this differs from other forms of global health engagement, especially for health security and noncommunicable diseases. How to provide appropriate coordination, the kind of leadership desired, and how to ensure the shared responsibility of funding beyond the traditional donors will all feature prominently. Global interconnectedness requires us to address the health of the planet’s entire population, irrespective of national borders. Engagement in global health is not simply a humanitarian concern but a priority for our collective well-being, efficient use of resources, and safeguarding our future.

Technical Appendix Infectious disease priorities, incidence, and commitments, The New Global Health.
