# Supplementary material for: The New Global Health
Source: Emerg Infect Dis. 2013 Aug;19(8):1192–7. doi: 10.3201/eid1908.130121 (PMC3739536; doi:10.3201/eid1908.130121)
Supplement: Technical Appendix — Infectious disease priorities, incidence, and commitments, The New Global Health. [file 13-0121-Techapp-s1.pdf]

Article DOI: http://dx.doi.org/10.3201/eid1908.130121

The New Global Health

Technical Appendix

Technical Appendix Table. Infectious disease priorities, incidence, and commitments, The New Global Health*

Disease	Incidence	Comments (References)
HIV/AIDS	34 million prevalent HIV infections; 2.5 million incident HIV infections; 1.7 million deaths, all in 2012; DALYs: 1,184/100,000 in 2010	Global targets and commitments<br>1) MDG 6: stopped by 2015 and begun to reverse spread of HIV/AIDS; achieve by 2010 universal access to treatment for HIV/AIDS for all persons who need it<br>2) Political Declaration on HIV and AIDS: Intensifying Our Efforts to Eliminate HIV and AIDS, 2011; United Nations General Assembly, 65th Session, 65/277: Commit to accelerate efforts to achieve the goal of universal access to antiretroviral treatment for persons eligible on the basis of WHO HIV treatment guidelines that indicate timely initiation of quality-ensured treatment for its maximum benefit, with the target of working toward having 15 million persons with HIV receiving antiretroviral treatment by 2015, reducing sexual transmission of HIV by 50% by 2015, reducing transmission of HIV among persons who inject drugs by 50% by 2015, work toward elimination of mother-to-child transmission of HIV and substantially reducing AIDS-related maternal deaths by 2015<br>3) Global Plan Toward the Elimination of New HIV Infections Among Children by 2015 and Keeping Their Mothers Alive, 2011–2015 Global Target:<br>1) Reduce the number of new HIV infections among children by 90%<br>2) Reduce the number of AIDS-related maternal deaths by 50%<br>3) PEPFAR: 6 million persons receiving antiretroviral therapy by the end of 2013, 1.5 million pregnant women provided prophylactic antiretroviral therapy, 4.7 million medical male circumcisions in eastern and southern Africa, and 1 billion condoms distributed (*1,2*)
Tuberculosis	8.7 million new cases, 1.4 million deaths in 2011, 13% of cases are co-infected with HIV, and 25% of TB deaths are HIV associated; XDR-TB has been reported in 84 countries, representing ≈9% of reported MDR-TB DALYs: 717/100,000 in 2010	Global targets and commitments: MDG 6: stop and reverse the TB epidemic by 2015 (compared with 1990) (achieved); The Global Plan to Stop TB, 2011–2015; Stop TB Partnership: By 2015, reduce prevalence and death rates by 50%, compared with their levels in 1990; By 2050, eliminate TB as a public health problem, defined as a global incidence of active TB <1 case/1 million population/y (*2,3*).
Malaria	219 million cases; 660,000 deaths in 2010; DALYs: 1,200/100,000 in 2010	Global targets and commitments: MDG to be stopped by 2015 and begun to reverse incidence of malaria and other major diseases; World Health Assembly, Roll Back Malaria: 75% reduction in case incidence by 2015 (50 countries on track). A total of 80% cases and deaths occur (2010 estimates) in 17 and 14 countries, respectively; Nigeria, Democratic Republic of Congo, and India account for 40% of cases. Malaria is considered endemic to 104 countries; 99 have ongoing transmission (*2,4*).
Neglected tropical diseases	152,000 deaths in 2010; DALYs: 378/100,000 in 2010	As defined by WHO, the term covers 17 diseases (including dengue, which is also considered separately in this table); milestones and targets are defined in the WHO report (*5*). The target of the US Government Global Health Initiative is to reduce the prevalence of 7 neglected tropical diseases by 50% among 70% of the affected population (contributing to the elimination of onchocerciasis in Latin America); elimination of lymphatic filariasis globally; and elimination of blinding trachoma and leprosy (*2,5–7*).

Disease	Incidence	Comments (References)
Selected major epidemic-prone and vaccine-preventable diseases		
Dengue	50–100 million cases/y; 20,000 deaths; DALYs 12/100,000 in 2010	Global targets and commitments: reduce dengue mortality rate by ≥50% by 2020, reduce dengue mortality rate by ≥25% by 2020, and estimate the true incidence of the disease by 2015 (2010 is used as a baseline). Dengue is the most widespread of all mosquito-borne viral infections, with a 30-fold increase in incidence over past 50 y. Almost half of the world's population is at risk in >100 dengue-endemic countries. The Asia–Pacific region is most heavily affected, but spread to new areas is occurring (*2,8*).
Yellow fever	200,000 cases/y; 30,000 deaths; DALYs <5/100,000 in 2010	Global targets and commitments: Inclusion of yellow fever vaccine in national childhood routine immunization programs administered at 9 mo of age, combined with the implementation of mass preventive vaccination campaigns to rapidly increase the population's immunity and to protect susceptible older age groups in selected high risk areas. Yellow fever is endemic to 45 countries of tropical Africa and Latin America and 900 million persons are at risk. The Yellow Fever Initiative is a joint collaboration of WHO and UNICEF, with the participation of national governments, and supported by the GAVI Alliance. Key partners include global public health agencies, industry, and others. It aims to prevent yellow fever epidemics in targeted countries through support for routine immunization, preventive mass vaccination, surveillance and outbreak response capacity, boosting vaccine production, and ensuring adequate vaccine stockpiling (*2,9*).
Cholera	3–5 million cases/y; 100,000–200,000 deaths; DALYs 65/100,000 in 2010	Global targets and commitments: Most relevant is MDG 7 (Ensure Environmental Sustainability), Target 7C: Halve by 2015 the proportion of the population without sustainable access to safe drinking water and basic sanitation. Substantial underreporting occurs (178–589,000 were reported annually to WHO over the recent 5 y-period). Recent developments include antibacterial agents for patients with moderate and severe dehydration (with rehydration), use of zinc, and increased use of oral cholera vaccine (*2,10,11*).
Typhoid	21.6 million cases/y; 190,000 deaths; DALYs 178/100,000 in 2010	Most relevant is MDG 7, as above (*2,7,12*).
Viral hepatitis (all forms)	500 million affected (350 million carriers of hepatitis B, 150 million carriers of hepatitis C); 1 million deaths/y; 57% cases of cirrhosis and 78% cases of hepatocellular carcinoma result from chronic hepatitis B or C virus infections; 1.4 million infections/y with hepatitis A; DALYs for all hepatitis: 192/100,000 in 2010	Global targets and commitments: Comprehensive approach to prevention and control of viral hepatitis: to prevent transmission, identify and treat those persons most at risk, integrate proven public health strategies for preventing viral hepatitis across the health system, and develop new vaccines and technologies for use in viral hepatitis prevention (*2,13*).
Polio	222 cases (wild poliovirus only) in 2012	Global targets and commitments: Poliovirus detection and interruption of transmission, the working target being to stop all wild poliovirus transmission by the end of 2014; strengthening routine immunization programs and withdrawal of oral polio vaccine, the primary target being the withdrawal of the type 2 component of oral polio vaccine in all routine immunization programs by mid-2016; containment and certification, the primary target being the certification of all 6 WHO regions as having eradicated all wild polioviruses by the end of 2018; and legacy planning, the initial target being to have a legacy strategy in place by the end of 2015 (*14,15*).

Disease	Incidence	Comments (References)
Bacterial meningitis (pneumococcal, *Haemophilus influenzae* type B, meningococcal)	276,000 deaths in 2010; DALYs: 287/100,000 in 2010	Global targets and commitments: Global Vaccine Action Plan: “improve health by extending by 2020 and beyond the full benefits of immunization to all people, regardless of where they are born, who they are, or where they live.” Specific goals: Achieve a world free of poliomyelitis; meet vaccination coverage targets in every region, country, and community; exceed the MDG4 target for reducing deaths of children; meet global and regional elimination targets; and develop and introduce new and improved vaccines and technologies (*2,7,16*).
Influenza	5%–10% annual attack rate globally in adults; 20%–30% in children; 508,000 deaths in 2010; DALYs: 279/100,000 in 2010	WHO advises that country-specific information about risk groups, disease incidence, and cost-effectiveness are needed to aid national policy makers and health program planners in making informed decisions about target groups and timing for vaccination. For countries considering initiation or expansion of programs for seasonal influenza vaccination, WHO recommends that pregnant women should have the highest priority. Additional risk groups to be considered for vaccination, in no particular order of priority, are children 6–59 mo of age, the elderly, persons with specific chronic medical conditions, and health care workers (*2,7,17*).

*DALYS, disability-adjusted life years; MDG, Millenium Development Goal; WHO, World Health Organization; PEPFAR, President’s Emergency Plan for AIDS Relief; MDR TB, multidrug-resistant tuberculosis, XDR extensively drug-resistant tuberculosis; TB, tuberculosis; UNICEF, United Nations Children’s Fund; GAVI, Global Alliance for Vaccines and Immunisation.

References

1. UNAIDS Report on the Global AIDS Epidemic. New York: UNAIDS; 2012.

2. Murray CJ, Vos T, Lozano R, Naghavi M, Flaxman AD, Michaud C, et al. Disability-adjusted life years (DALYs) for 291 diseases and injuries in 21 regions, 1990–2010: a systematic analysis for the global burden of disease study 2010. Lancet. 2012;380:2197–223. PubMed http://dx.doi.org/10.1016/S0140-6736(12)61689-4

3. Global Tuberculosis Report WHO. 2012 [cited 2103 May 16]. http://apps.who.int/iris/bitstream/10665/75938/1/9789241564502_eng.pdf

4. Report WM. 2012 [cited 2013 May 16]. http://www.who.int/malaria/publications/world_malaria_report_2012/wmr2012_full_report.pdf

5. World Health Organization. 2012. Accelerating work to overcome the global impact of neglected tropical diseases [cited 2013 May 16]. http://www.who.int/neglected_diseases/NTD_RoadMap_2012_Fullversion.pdf

6. US Global Health Initiative [cited 2013 May 16]. http://www.ghi.gov/about/goals/index.htm

7. Lozano R, Naghavi M, Foreman SL, Lim S, Shibuya K, Aboyans V, et al. Global and regional mortality from 235 causes of death for 20 age groups in 1990 and 2010: a systematic analysis for the Global Burden of Disease Study 2010. Lancet. 2012;380:2095–128. PubMed http://dx.doi.org/10.1016/S0140-6736(12)61728-0

8. World Health Organization. 2012. Global strategy for dengue prevention and control, 2012–2020 [cited 2013 May 16]. http://apps.who.int/iris/bitstream/10665/75303/1/9789241504034_eng.pdf

9. World Health Organization. 2010. Yellow fever initiative. Providing an opportunity of a lifetime [cited 2013 May 16]. http://www.who.int/csr/disease/yellowfev/YFIbrochure.pdf

10. Waldman RJ, Mintz ED, Papowitz HE. The cure for cholera: improving access to safe water and sanitation. N Engl J Med. 2013;368:592–4. PubMed http://dx.doi.org/10.1056/NEJMp1214179

11. World Health Organization. Cholera [cited 2013 May 16]. http://www.who.int/features/factfiles/cholera/en/index.html

12. Crump JA, Luby SP, Mintz ED. The global burden of typhoid fever. Bull World Health Organ. 2004;82:346–53. PubMed

13. World Health Organization. 63rd World Health Assembly. Viral hepatitis [cited 2013 May 16]. http://apps.who.int/gb/ebwha/pdf_files/WHA63/A63_15-en.pdf

14. The Global Polio Emergency Action Plan 2012–2013 [cited 2013 May 16]. http://www.polioeradication.org/Portals/0/Document/Resources/StrategyWork/EAP_201205.pdf

15. Polio eradication and endgame strategic plan 2013–2018 [cited 2013 May 16]. http://www.polioeradication.org/Portals/0/Document/Resources/StrategyWork/EndGameStratPlan_20130123_ENG.pdf

16. World Health Organization. 2013. Global vaccine action plan 2011–2020 [cited 2013 May 16]. http://www.who.int/immunization/global_vaccine_action_plan/GVAP_doc_2011_2020/en/index.html

17. World Health Organization. Vaccines against influenza. WHO position paper, November 2012. Wkly Epidemiol Rec. 2012;87:461–76. PubMed
